# Characterizing CRP dynamics during acute infections

**DOI:** 10.1007/s15010-024-02422-7

**Published:** 2024-10-28

**Authors:** Stacey S. Cherny, Rafael Y. Brzezinski, Asaf Wasserman, Amos Adler, Shlomo Berliner, Daniel Nevo, Saharon Rosset, Uri Obolski

**Affiliations:** 1 Department of Epidemiology and Preventive Medicine, School of Public Health, Faculty of Medical & Health Sciences Tel Aviv University, Tel Aviv, Israel; 2 Department of Environmental Studies, Porter School of the Environment and Earth Sciences, Faculty of Exact Sciences Tel Aviv University, Tel Aviv, Israel; 3 Faculty of Medical & Health Sciences, Tel Aviv University, Tel Aviv, Israel; 4 Internal Medicine “C” and “E”, Tel Aviv Sourasky Medical Center, Tel Aviv, Israel; 5 Department of Statistics and Operations Research, Faculty of Exact Sciences, Tel Aviv University, Tel Aviv, Israel

**Keywords:** CRP dynamics, CRP trajectories, Bacteremia, Infection, Antibiotics

## Abstract

**Purpose:**

C-reactive protein (CRP) is a common proxy of inflammation, but accurate characterizations of its dynamics during acute infections are scant. The goal of this study was to examine C-reactive protein (CRP) trajectories in hospitalized patients with viral infections, confirmed bacteremia (stratified by Gram-negative or Gram-positive bacteria), and non-bacteremic infections/inflammations, considering antibiotic treatment.

**Methods:**

Electronic medical records from Tel Aviv Sourasky Medical Center (July 2007-May 2023) were analyzed. Patients with blood cultures or positive viral tests were included. CRP levels were modeled using generalized additive mixed-effects models (GAMMs) and observed up to 150 h after initial infection diagnosis. Patients with initial CRP levels > 31.9 were excluded, to remove individuals already in a highly active inflammatory process. The shapes of the CRP curves were characterized and peak CRP as well as area under the CRP curve were the primary variables of interest.

**Results:**

Viral infections had the lowest and flattest CRP curves. Non-bacteremic infections showed intermediate levels, while bacteremia (especially Gram-negative under antibiotic treatment) had the highest CRP peaks. For instance, peak CRP ranged from 15.4 mg/L in viral infections without antibiotics to 140.9 mg/L in Gram-negative bacteremia with antibiotics.

**Conclusions:**

CRP trajectories significantly differ based on infection type and antibiotic treatment. Frequent CRP measurement could be a valuable diagnostic and risk stratification tool in hospitalized patients.

**Supplementary Information:**

The online version contains supplementary material available at 10.1007/s15010-024-02422-7.

## Introduction

Inflammation is the body’s fundamental response to infection. C-reactive protein (CRP) is a routinely measured biomarker, serving as a common proxy of inflammation in clinical settings [1]. CRP levels tend to increase following infection, and as a result are generally employed as an indicator of severity of infection. Initial CRP levels can also suggest whether the infection is bacterial, which tends to result in higher levels, or viral [1–5]. Nonetheless, despite CRP’s utility and ubiquity, there are no accurate characterizations of CRP dynamics, over time, during acute infections.

In the present study, we examined the trajectory of CRP in hospitalized patients who have either had a confirmed bacteremia, a confirmed viral infection, or a non-bacteremic infection/inflammation. We particularly focussed on differences in the peaks of these curves as well as the area under them (AUC) as the main summary statistics.

## Methods

### Data

In this single-center retrospective cohort study, we extracted electronic medical records of all patients admitted to Tel Aviv Sourasky Medical Center, Israel, who had blood cultures drawn, or tested positive for a viral infection, made available between July 2007 and May 2023.

The outcome was patients’ CRP levels across time, considered in mg/L throughout. The time of CRP measurement was defined relative to each patient’s time zero: the time of the first bacterial blood culture or viral diagnostic test collection, and was measured up to 24 h prior to culture or viral test obtainment, and up to 150 h following it. Patients whose initial CRP level was higher than 31.9 were excluded. This exclusion criterion, constituting three standard deviations above normal CRP levels [6], was devised to remove individuals already in a highly active inflammatory process, so that standard CRP dynamics could be captured.

Data were stratified into three cohorts for the analysis (Supplementary Table 1): (1) a subsample of patients who had negative bacterial blood cultures (35,975 patients, 103,333 CRP measurements) and had not tested positive for viral infection, but likely had some infection/inflammation leading to drawing of culture; (2) patients who tested positive for any virus (Supplementary Table 2), while testing negative for bacteremia (1805 patients, 4426 CRP measurements); (3) a subsample of patients who had positive bacterial growth in their blood cultures. Cultures with organisms likely to be contaminants were excluded (Supplementary Tables 3–4, respectively). Patients who had growth of non-contaminant bacteria in any of the cultures drawn were stratified to those who only had a Gram-negative infection (718 patients, 2658 CRP measurements) and those who only had a Gram-positive infection (378 patients, 1473 CRP measurements). All patients were further classified as to whether they received any antibiotic treatment up to 24 h before or after time zero vs. either not having received antibiotics or having received them outside this window. Additional covariates included in analyses were patients’ Charlson comorbidity index (CCI), sex, and age.

### Statistical analysis

We employed generalized additive mixed-effects models (GAMMs), using the R package mgcv [7], as our primary method of analysis. GAMMs are an extension to generalized linear mixed-effects models, where the assumption of a linear relationship of a covariate and the outcome is replaced with a smooth estimation through splines. These models allowed for flexible estimation of the relationship between the time and CRP trajectory, while controlling for age, sex, and CCI, and including a random intercept to account for patients’ repeated measures. A GAMM was fitted to each of the cohorts. For the third cohort, bacterial Gram stain was included as an additional covariate. In addition, because the sample of patients who simultaneously tested negative for both bacterial and viral infection was very large, we randomly selected 1000 patients who received antibiotic treatment within the time window and 1000 who did not - numbers chosen to be relatively comparable in size to the virus cohort.

While the GAMMs produce inferential statistics for the smooth terms and the fixed effects coefficients, we were also interested in testing whether the peak CRP observed, as well as the area under the CRP curve (AUC-CRP), differed between strata. Statistical inference (confidence interval and p-value calculations) was done by bootstrap analysis, computing both the peak CRP and the area under the curve within the first 72 h of observation in each of 1000 bootstrap samples for each of the analyses performed. Statistical significance was determined at a *p* < 0.05 level. Further details are in Supplementary Tables 5–6.

## Results

**Fig. 1:**
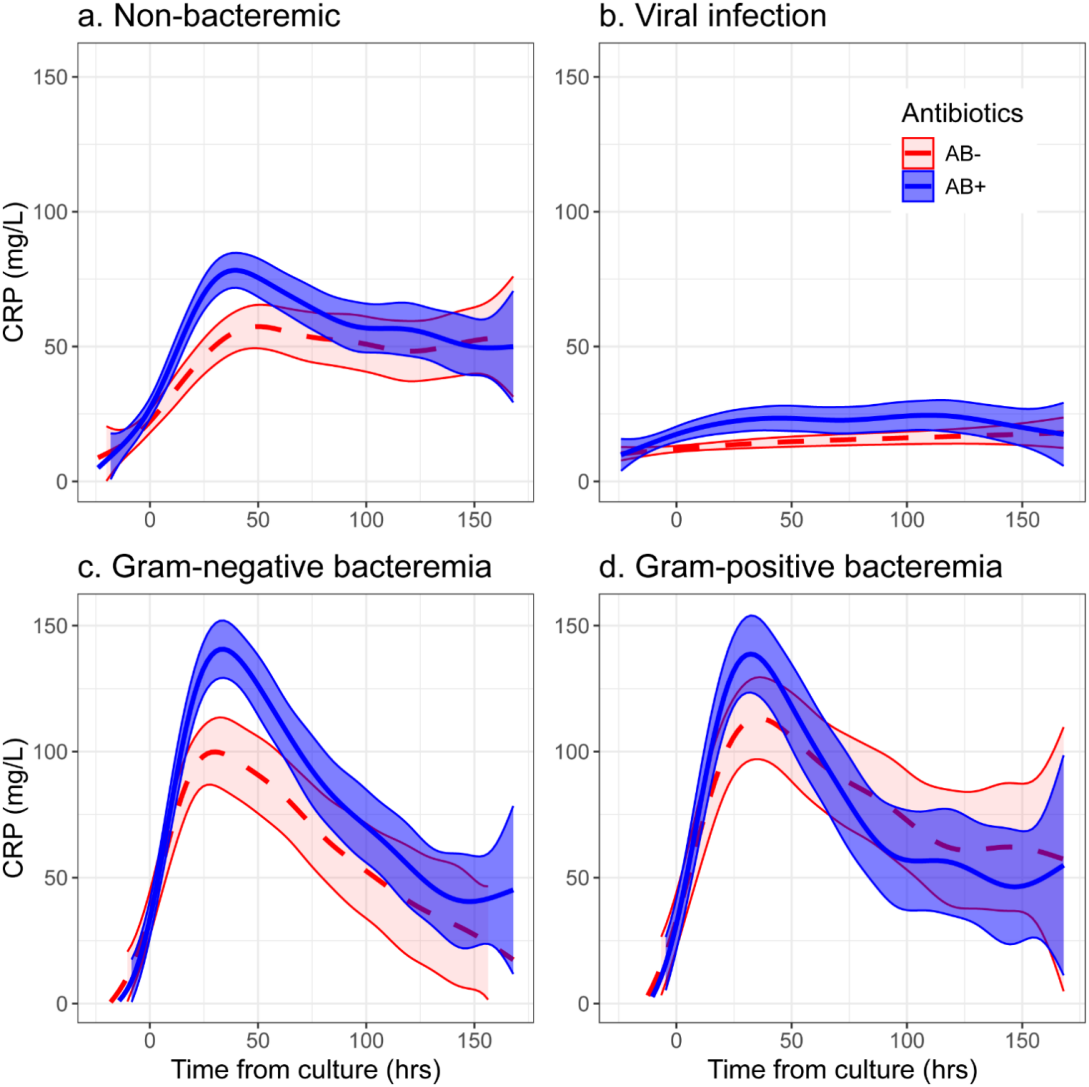
Estimated trajectory of CRP, stratified by exposure to antibiotics at up to 24 h before or after time zero (AB + vs. AB-), in four cohorts. The vertical axes represent CRP in mg/L, whereas the horizontal axis is the hours from time zero, which is the time of bacterial culture (**a**, **c**, **d**) or viral infection test (**b**). (**a**) Patients who tested negative for a viral infection and negative for bacteremia. (**b**) Patients who tested positive for a viral infection and negative for bacteremia. (**c**) Patients who tested positive only for a Gram-negative bacteremia. (**d**) Patients who tested positive only for a Gram-positive bacteremia. CRP trajectories were estimated via generalized additive models, and plotted while accounting for a parametric term for antibiotic use. The models were also adjusted for patients’ age, sex and Charlston comorbidity index. Bands around the curves represent 95% confidence intervals

We compared the estimated trajectories of CRP across the cohorts described above, while controlling for patients’ age, sex, and CCI, and stratifying by presence of antibiotic treatment (AB-, AB+, respectively; Fig. 1). First, we observed that the viral cohort’s dynamics stood out with low values and relatively flat curves (peak CRP AB- 15.4, 95%CI [13.3, 17.7]; AB + 24.5 [20.0, 31.1]; Fig. 1.B). The bacteremia-negative cohort had intermediate CRP levels (peak CRP AB- 57.4 [49.3, 65.3]; AB + 77.9 [70.8, 83.7]; Fig. 1.A), but still retained a similar pattern to the bacteria positive cohorts (peak CRP AB-/Gram- 101.8 [93.3, 110.9]; AB+/Gram- 140.9 [130.7, 151.4]; AB-/Gram + 114.6 [98.5, 130.5]; AB+/Gram + 138.9 [126.1, 152.9]; Fig. 1.C–D).

We further tested differences between CRP trajectories across the four cohorts, both between those who received antibiotics and for those who did not (Supplementary Tables 5–6). With the exception of differences between Gram-negative and Gram-positive cohorts, all cross-cohort comparisons yielded statistically significant differences, both when examining differences between CRP peaks or AUC-CRP. That is, for both the antibiotic treatment and no treatment groups, CRP peaks and AUCs were higher for those who had bacteremia (Gram-negative or Gram-positive) vs. those who had either a viral infection (e.g., Gram-negative/AB- peak CRP difference 84.5 [78.4, 96.0]) or were bacteremia-negative (e.g., Gram-negative/AB- peak CRP difference 42.5 [32.9, 56.1]). The differences between CRP peaks and AUCs of the Gram-negative and Gram-positive cohorts were not statistically significant either for those who received antibiotics or for those who did not.

Finally, we tested whether the differences in CRP levels between the antibiotic treatment strata varied between cohorts (second sections of Supplementary Tables 5–6); i.e., whether there is an interaction between the infection type and antibiotic treatment. Briefly, this difference was largest in the Gram-negative cohort, comparable in the bacteremia-negative and Gram-positive cohorts, and lowest in the virus cohort. The Gram-positive cohort exhibited high variance, so comparisons to it were mostly non-significant. The AUC-CRP levels presented similar trends to the peak CRP levels, albeit with lower variance and more distinct differences.

## Discussion

In this study, we characterized the trajectories of CRP during acute infections, under bacteremia, viral infections, or no detected bacteremia. The curves were stratified by Gram stain and antibiotic use, while adjusted for age, sex, and CCI. These results provide for the first time, to the best of our knowledge, explicit CRP curves based on a large cohort. We observed marked differences in the CRP curves’ shapes, peaks, and AUCs, between the cohorts and following antibiotic use.

The receipt of antibiotics was associated with increased CRP levels across all cohorts. This could occur due to several reasons. First, there might be confounding by indication, as patients “worse-off” are more likely to receive antibiotic treatment. However, we constrained the initial CRP levels to “normal” ranges, resulting in similar CRP levels close to time zero. The models were also adjusted for age and CCI, potentially mitigating such an effect. Furthermore, it has been shown that CRP’s half-life is unaffected by comorbidities, and hence the pathological process underlying CRP synthesis, such as infection, should be the determining factor of its concentration [8]. Second, there might be a causal effect of antibiotics on CRP levels, through their effect on the patient or the bacteria. If this is the case, we would not expect to see this phenomenon during viral or bacteremia-negative infections. However, bacterial cultures have low yields [9] and thus the antibiotic treatment could still have affected individuals with a false-negative diagnosis of no bacterial infection in both non-bacteremic cohorts. A causal reason for these differences could be the “unblocking” of immune paresis, that was imposed by the invading organism on the patients’ innate immune system. Alternatively, the antibiotics could cause the release of “inflammogenic” molecules from the bacterial capsule. Or, inflammation could increase due to clearance of degraded bacteria from the body. Potentially, these could be exacerbated by Gram-negative infections, due to their endotoxins, in a phenomenon analogous to the Jarisch-Herxheimer reaction [10]. Although the causal nature of this phenomenon is yet to be determined, the estimated curves still reliably represent CRP trajectories across the cohorts.

As implied by previous research based on either single or few measurements [11], CRP levels were substantially lower in the viral cohort. However, the bacteremia-negative cohort exhibited surprisingly high CRP levels. These could again be due to the low yields of bacterial cultures or confounding, as described above. The Gram stains of bacteria were weakly associated with differences in CRP levels, mainly when examining the AUC-CRP. Further research, ideally prospective, is needed to establish the relationship between bacteria’s Gram stain and patients’ CRP levels.

To conclude, different acute infections and antibiotic treatment are associated with distinct CRP trajectories. Frequent measurement of this low cost, real-time inflammatory marker should be further explored as a diagnostic and risk stratification tool in hospitalized patients.

## Electronic supplementary material

Below is the link to the electronic supplementary material.

Supplementary Material 1

## Data Availability

The data pertain to the patients’ electronic medical records. These are private and cannot be shared without approval from Tel Aviv Sourasky Medical Center’s IRB. Upon request, the authors and the individuals interested in accessing the data can write a formal request to the aforementioned IRB and seek its approval.
