# Supplementary material for: Characterizing CRP dynamics during acute infections
Source: Infection. 2024 Oct 28;53(3):1199–203. doi: 10.1007/s15010-024-02422-7 (PMC12137512; doi:10.1007/s15010-024-02422-7)
Supplement: Supplementary file 1 — Supplementary Material 1 [file 15010_2024_2422_MOESM1_ESM.docx]

# Supplementary material

## Methods

GAMMs are an extension to generalized linear mixed-effects models, where the assumption of a linear relationship of a covariate and the outcome is replaced with a smooth estimation through splines. While the GAMMs produce inferential statistics of the fixed effects coefficients, we were interested in testing whether the peak CRP observed, as well as the area under the CRP curve (AUC-CRP), differed between strata. Statistical inference (confidence interval and p-value calculations) was done by bootstrap analysis, computing both the peak CRP and the area under the curve within the first 72 hours of observation in each of 1000 bootstrap samples for each of the analyses performed. Statistical significance was determined at a p<0.05 level.

| **Supplementary Table 1: Sample sizes corresponding to Figure 1, in terms of CRP measurements and patients.** | | | | |
| --- | --- | --- | --- | --- |
|  | **Number of CRP measurements** | | **Number of patients** | |
|  | AB- | AB+ | AB- | AB+ |
| Panel a* | 2586 | 3686 | 1000 | 1000 |
| Panel b | 3315 | 1111 | 1455 | 350 |
| Panel c | 1062 | 1596 | 378 | 340 |
| Panel d | 658 | 815 | 200 | 178 |

*Because the sample of patients who simultaneously tested negative for both bacterial and viral infection was very large, we randomly selected 1000 patients who received antibiotic treatment within the time window and 1000 who did not - numbers chosen to be relatively comparable in size to the virus cohort.

| **Supplementary Table 2: Total number of times each species was detected in the virus positive sample.** | |
| --- | --- |
| **Test** | **Count** |
| Adeno Virus RT-PCR | 50 |
| Coxsackievirus Ab-B | 15 |
| EBV (Epstein-Barr virus) PCR_ quantitative - blood | 2 |
| Entero-Echo Virus | 12 |
| Enterovirus FilmArray | 17 |
| Enterovirus PCR - CSF | 81 |
| Enterovirus PCR - feces | 5 |
| Enterovirus RT-PCR | 17 |
| Epstein Barr Virus IgM | 165 |
| Human metapneumovirus RT-PCR | 47 |
| Human Rhinovirus / Enterovirus FilmArray | 2 |
| Influenza A - blood | 2 |
| Influenza A Ag - nasal fluid | 11 |
| Influenza A FilmArray | 2 |
| Influenza A RT-PCR | 16 |
| Influenza A RT-PCR - nasal fluid | 64 |
| Influenza A1 RT-PCR Ct- nasal fluid | 3 |
| Influenza B - blood | 7 |
| Influenza B Ag - nasal fluid | 11 |
| Influenza B FilmArray | 2 |
| Influenza B RT-PCR | 9 |
| Influenza B RT-PCR - nasal fluid | 62 |
| INFLUENZA VIRUS RT-PCR | 130 |
| Measles (rubeola) virus PCR | 6 |
| Measles Ab_ IgM-B | 37 |
| Monkeypox PCR result | 20 |
| Mumps Ab_ IgM-B | 29 |
| Parainfluenza 1 Ag detection- Nasal fluid | 10 |
| Parainfluenza 2 Ag detection- Nasal fluid | 10 |
| Parainfluenza 3 Ag detection- Nasal fluid | 10 |
| Parainfluenza RT-PCR | 40 |
| Parainfluenza type 1_ RT-PCR | 7 |
| Parainfluenza type 2_ RT-PCR | 7 |
| Parainfluenza type 3_ RT-PCR | 3 |
| Parainfluenza type 4_ RT-PCR | 7 |
| Parvovirus B19 IgM-B | 77 |
| Respiratory syncytial virus RT-PCR | 173 |
| Rhinovirus RT-PCR | 36 |
| RSV (Respiratory Syncytial Virus) FilmArray | 2 |
| RSV antigen - nasal aspiration | 13 |
| RSV_ respiratory syncytial virus_ Ab - blood | 1 |
| Rubella_ IgM Ab - blood | 9 |
| SIMPLEXA FLU A/B & RSV Control | 51 |
| Varicella-zoster Ab_ IgM-B | 114 |
| Varicella-zoster IgM-Ct | 11 |
| Varicella zoster PCR | 857 |
| Varicella zoster PCR - CSF | 92 |
| varicella zoster virus_ identification with biopsy | 347 |
| West Nile virus IgM - blood | 45 |
| West Nile virus IgM - CSF | 18 |
| **Total** | **2764** |

| **Supplementary Table 3: Total number of times each non-contaminant species was detected in the Gram-negative sample.** | |
| --- | --- |
| **Species** | **Count** |
| Achromobacter xylosoxidans | 2 |
| Acinetobacter baumannii | 1 |
| Aeromonas sobria | 1 |
| Bacillus species | 2 |
| Brucella melitensis | 4 |
| Brucella spp | 2 |
| Candida parapsilosis | 1 |
| Citrobacter freundii | 8 |
| Citrobacter koseri | 20 |
| Coagulase negative Staphylococcus | 15 |
| Comamonas testosteroni | 1 |
| Corynebacterium species | 1 |
| Enterobacter cloacae | 8 |
| Enterobacter cloacae complex | 19 |
| Enterobacter hormaechei | 1 |
| Enterococcus casseliflavus | 1 |
| Escherichia coli | 608 |
| Gram negative bacilli | 401 |
| Gram negative coccobacilli | 1 |
| Haemophilus influenzae | 10 |
| Haemophilus influenzae IV | 1 |
| Haemophilus influenzae V | 1 |
| Haemophilus influenzae VI | 1 |
| Haemophilus influenzae,not type B | 2 |
| Haemophilus spp | 1 |
| Klebsiella aerogenes | 9 |
| Klebsiella oxytoca | 21 |
| Klebsiella pneumoniae | 87 |
| Klebsiella pneumoniae ssp pneumoniae | 91 |
| Moraxella (Branhamella) catarrhalis | 2 |
| Moraxella group | 1 |
| Morganella morganii | 1 |
| Neisseria meningitidis | 1 |
| Neisseria spp | 1 |
| Pasteurella multocida | 2 |
| Proteus mirabilis | 2 |
| Providencia stuartii | 1 |
| Pseudomonas aeruginosa | 90 |
| Pseudomonas putida | 2 |
| Pseudomonas spp | 39 |
| Salmonella enteritidis | 4 |
| Salmonella group | 2 |
| Salmonella group D | 4 |
| Salmonella ser. Typhi | 2 |
| Salmonella ser.Paratyphi A | 3 |
| Salmonella spp | 6 |
| Serratia marcescens | 19 |
| Staphylococcus hominis | 2 |
| Staphylococcus hominis ssp hominis | 1 |
| Staphylococcus simulans | 1 |
| Streptococcus constellatus | 1 |
| Streptococcus cristatus | 1 |
| Streptococcus mitis/Streptococcus oralis | 2 |
| Streptococcus salivarius | 1 |
| Streptococcus viridans group | 4 |
| Yeast | 1 |
| **Total** | **1517** |

| **Supplementary Table 4: Total number of times each non-contaminant species was detected in the Gram-positive sample.** | |
| --- | --- |
| **Species** | **Count** |
| Acinetobacter baumannii | 2 |
| Aerococcus viridans | 2 |
| Coagulase negative Staphylococcus | 18 |
| Coagulase positive Staphylococcus | 34 |
| Corynebacterium species | 1 |
| Enterococcus faecalis | 82 |
| Enterococcus faecium | 10 |
| Gram negative bacilli | 1 |
| Gram positive cocci consistent with Pneumococci | 4 |
| Group D Streptococcus | 31 |
| Providencia stuartii | 1 |
| Staphylococcus spp | 2 |
| Staphylococcus aureus | 148 |
| Staphylococcus epidermidis | 1 |
| Staphylococcus haemolyticus | 2 |
| Staphylococcus hominis | 2 |
| Staphylococcus hominis ssp hominis | 1 |
| Staphylococcus lugdunensis | 2 |
| Staphylococcus saprophyticus | 12 |
| Staphylococcus sciuri | 1 |
| Stenotrophomonas maltophilia | 1 |
| Streptococcus-beta haemolytic not group A | 2 |
| Streptococcus agalactiae | 77 |
| Streptococcus anginosus | 19 |
| Streptococcus b-hemolyticus | 98 |
| Streptococcus dysgalactiae ssp dysgalactiae | 23 |
| Streptococcus dysgalactiae ssp equisimilis | 28 |
| Streptococcus gallolyticus | 6 |
| Streptococcus gallolyticus ssp pasteurianus | 14 |
| Streptococcus group G | 2 |
| Streptococcus mitis/Streptococcus oralis | 3 |
| Streptococcus mutans | 4 |
| Streptococcus pneumoniae | 36 |
| Streptococcus pyogenes | 71 |
| Streptococcus salivarius | 1 |
| Streptococcus spp | 2 |
| Streptococcus viridans group | 7 |
| **Total** | **751** |

| **Supplementary Table 5: Estimates of differences in maximum CRP from time 0 until 72 hours. 95% confidence intervals were obtained via 1000 repetitions of BCa bootstrapping. Two-tailed empirical p-values were computed under percentile bootstrapping with the assumption that the bootstrap distribution is the same as the null distribution, up to a mean shift. The three corresponding R-squared values for the three GAMM models are presented in the rightmost column.** | | | | | | |
| --- | --- | --- | --- | --- | --- | --- |
|  | **Comparison** | **CRP peak difference** | **95% CI** | | **p-value** | **R-squared** |
| Difference between AB- vs AB+ within panels | Panel a - Bact- | 20.9 | 10.7 | 30.0 | 0.001 | 0.542 |
|  | Panel b - Virus | 8.0 | 4.5 | 16.0 | 0.008 | 0.497 |
|  | Panel c - Gram- | 40.8 | 26.0 | 52.8 | 0.001 | 0.641 |
|  | Panel d - Gram+ | 25.5 | 2.1 | 44.3 | 0.007 |  |
| Difference between differences across panels | c vs d | -15.3 | -39.5 | 9.0 | 0.112 |  |
|  | b vs c | 32.7 | 15.3 | 43.4 | 0.001 |  |
|  | b vs d | 17.4 | -6.9 | 37.2 | 0.054 |  |
|  | a vs c | 19.9 | 1.7 | 35.2 | 0.014 |  |
|  | a vs d | 4.6 | -20.0 | 25.9 | 0.512 |  |
|  | a vs b | -12.8 | -22.4 | -0.4 | 0.010 |  |
| AB+ | c vs d | -1.9 | -19.2 | 14.9 | 0.668 |  |
|  | b vs c | 117.2 | 104.4 | 128.0 | 0.001 |  |
|  | b vs d | 115.3 | 100.5 | 129.7 | 0.001 |  |
|  | a vs c | 62.4 | 51.1 | 76.0 | 0.001 |  |
|  | a vs d | 60.5 | 46.0 | 75.4 | 0.001 |  |
|  | a vs b | -54.8 | -60.8 | -43.5 | 0.001 |  |
| AB- | c vs d | 13.4 | -7.2 | 30.1 | 0.432 |  |
|  | b vs c | 84.5 | 78.4 | 96.0 | 0.001 |  |
|  | b vs d | 97.9 | 82.5 | 114.9 | 0.001 |  |
|  | a vs c | 42.5 | 32.9 | 56.1 | 0.001 |  |
|  | a vs d | 55.8 | 38.4 | 74.2 | 0.001 |  |
|  | a vs b | -42.0 | -49.8 | -34.0 | 0.001 |  |

| **Supplementary Table 6: Estimates of differences in area under the CRP curve (AUC-CRP) from time 0 to 72 hours. 95% confidence intervals were obtained via 1000 repetitions of BCa bootstrapping. Two-tailed empirical p-values were computed under percentile bootstrapping with the assumption that the bootstrap distribution is the same as the null distribution, up to a mean shift.** | | | | | |
| --- | --- | --- | --- | --- | --- |
|  | **Comparison** | **AUC-CRP difference** | **95% CI** | | **p-value** |
| AB- vs AB+ | Panel a - Bact- | 1220.5 | 761.4 | 1618.4 | 0.001 |
|  | Panel b - Virus | 565.7 | 289.2 | 829.1 | 0.001 |
|  | Panel c - Gram- | 1977.4 | 1256.4 | 2551.8 | 0.001 |
|  | Panel d - Gram+ | 961.3 | -62.4 | 1939.5 | 0.031 |
| Difference between differences across panels | c vs d | -1016.1 | -2152.1 | 249.3 | 0.047 |
|  | b vs c | 1411.7 | 679.7 | 2088.6 | 0.001 |
|  | b vs d | 395.7 | -697.7 | 1368.1 | 0.234 |
|  | a vs c | 756.9 | -120.7 | 1498.1 | 0.030 |
|  | a vs d | -259.2 | -1397.6 | 784.4 | 0.397 |
|  | a vs b | -654.8 | -1159.0 | -94.8 | 0.009 |
| AB+ | c vs d | -362.0 | -1152.5 | 390.8 | 0.172 |
|  | b vs c | 6378.3 | 5848.5 | 6925.3 | 0.001 |
|  | b vs d | 6016.3 | 5366.9 | 6742.8 | 0.001 |
|  | a vs c | 3349.8 | 2823.3 | 3998.4 | 0.001 |
|  | a vs d | 2987.8 | 2352.7 | 3696.4 | 0.001 |
|  | a vs b | -3028.5 | -3383.9 | -2534.8 | 0.001 |
| AB- | c vs d | 654.1 | -317.6 | 1538.1 | 0.230 |
|  | b vs c | 4966.6 | 4497.7 | 5521.5 | 0.001 |
|  | b vs d | 5620.7 | 4840.1 | 6405.1 | 0.001 |
|  | a vs c | 2592.9 | 2089.2 | 3329.0 | 0.001 |
|  | a vs d | 3247.0 | 2392.3 | 4094.2 | 0.001 |
|  | a vs b | -2373.7 | -2690.5 | -1948.5 | 0.001 |
